# Understanding the Impact of Hierarchical Nanostructure in Ternary Organic Solar Cells

**DOI:** 10.1002/advs.201500250

**Published:** 2015-09-02

**Authors:** Jin Fang, Zaiyu Wang, Jianqi Zhang, Yajie Zhang, Dan Deng, Zhen Wang, Kun Lu, Wei Ma, Zhixiang Wei

**Affiliations:** ^1^ CAS Key Laboratory of Nanosystem and Hierarchical FabricationNational Center for Nanoscience and TechnologyBeijing100190P. R. China; ^2^ State Key Laboratory for Mechanical Behavior of MaterialsXi'an Jiaotong UniversityXi'an710049P. R. China; ^3^ University of Chinese Academy of SciencesBeijing100049P. R. China

**Keywords:** conjugated polymers, conjugated small molecules, hierarchical nanostructures, morphology, ternary organic solar cells

## Abstract

Ternary organic solar cells (OSCs), which blend two donors and fullerene derivatives with different absorption ranges, are a promising potential strategy for high‐power conversion efficiencies (PCEs). In this study, inverted ternary OSCs are fabricated by blending a highly crystalline small molecule BDT‐3T‐CNCOO in a low band gap polymer PBDTTT‐C‐T:PC_71_BM. As the small molecule is introduced, the overall PCEs increase from 7.60% to 8.58%. The morphologies of ternary blends are studied by combining transmission electron microscopy and X‐ray scattering techniques at different length scales. Hierarchical phase separation is revealed in the ternary blend, which is composed of domains with sizes of ≈88, ≈50, and ≈20 nm, respectively. The hierarchical phase separation balances the charge separation and transport in ternary OSCs. As a result, the fill factors of the devices significantly improve from 58.4% to 71.6%. Thus, ternary blends show higher hole mobility and higher fill factor than binary blends, which demonstrates a facile strategy to increase the performance of OSCs.

## 1 Introduction

Bulk heterojunction organic solar cells (OSCs) based on donor/acceptor bicontinuous interpenetrating networks provide a promising approach to light‐weight, highly flexible, and cost‐effective solar energy conversion.^1^, ^2^, ^3^, ^4^ To obtain higher power conversion efficiencies (PCEs), researchers focused on developing new materials with suitable energy levels and high carrier mobilities, optimizing the morphological characteristics of active layers, utilizing various interfacial modifications, and developing new device architectures.^5^, ^6^, ^7^, ^8^, ^9^, ^10^, ^11^, ^12^, ^13^, ^14^ Currently developed PCEs exceed 10% in single‐junction OSCs.^15^, ^16^ Ternary OSCs have been developed as an emerging candidate to achieve high PCEs in which two or more donors are mixed with different absorption spectra to enhance light harvesting in single‐junction OSCs.^17^, ^18^, ^19^, ^20^, ^21^, ^22^

The photo‐harvesting ability of ternary OSCs is improved by selecting the materials with complementary absorption spectra, thereby increasing short circuit current density (*J*_sc_). However, the excess of third component often has unfavorable influence on the phase separation and molecular ordering in the ternary blends.^18^, ^23^ Therefore, selecting the third component with structure compatibility is reported as a successful strategy to achieve high‐performance ternary OSCs.^21^, ^22^ Conjugated small molecule, which displays good crystallinity, high intrinsic carrier mobility, and good miscibility with the host polymer matrix, is one of the potential candidates for the ternary OSCs.^18^ Several successful small molecule/polymer/[6,6]‐phenyl‐C_71_‐butyric acid methyl ester (PC_71_BM) based ternary OSCs have reported that the small molecule incorporation improved the pathway for charge carrier and the morphology of the ternary film.^18^, ^24^, ^25^, ^26^ Recently, we have reported that the conjugated small molecules (BDT‐3T‐CNCOO) can largely increase fill factor (FF) of polymer solar cells by modifying the morphology, and ternary OSCs with an efficiency of 8.40% are obtained using a conventional device structure.^27^ The optical band gap of the polymer PBDTTPD‐HT selected in the work is as high as 1.86 eV. So it is interesting to study whether the small molecule BDT‐3T‐CNCOO can be used in the other systems, especially a low band gap conjugated polymer. On the other hand, comparing the donor–acceptor bicontinuous nanomorphology in binary blends, the phase behavior after the incorporation of the third component in ternary blend is not clear yet due to the low contrast among different components.

In this study, a ternary OSC with an average PCE of 8.58% is developed by incorporating a high ratio of conjugated small molecules into a low band gap polymer solar cell PBDTTT‐C‐T:PC_71_BM binary system. The PCE of OSC is enhanced from 7.60% in binary OSC to 8.58% in ternary OSC because FF is increased from 58.4% to 71.6%. Morphological characteristics, i.e. crystallinity, and phase separation of ternary blend films are revealed by transmission electron microscopy (TEM), grazing‐incidence wide‐angle X‐ray scattering (GIWAXS), and resonant soft X‐ray scattering (R‐SoXS) at different length scales. The optimal active layer displays a hierarchical phase separation, which possesses better crystallinity, purer domains, and multiscale domain sizes. As a result, ternary blends show higher hole mobility and higher FF than binary blends, which ascribe to the higher PCE of ternary OSCs.

## 2 Results and Discussion

### 2.1 Molecular Structure and Property

**Figure 1:**
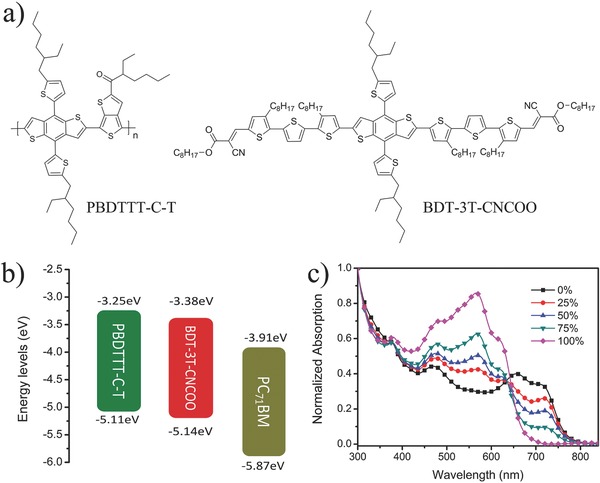
a) Chemical structures of the polymer PBDTTT‐C‐T and the small molecule BDT‐3T‐CNCOO. b) Energy band diagram of the ternary components. c) UV–vis absorption spectra of the active layer with different ratios of BDT‐3T‐CNCOO.

PBDTTT‐C‐T is a typical low band gap polymer with good device performance, and a 9.13% PCE have been reported based on inverted structure with a cross‐linkable water/alcohol‐soluble conjugated polymer interlayer (PFN‐OX), however, FF was 67% in the best system of PBDTTT‐C‐T and was approximately 60% in the other device structures.^14^, ^28^, ^29^ And it show much promising to get higher efficiency of the PBDTTT‐C‐T, not only this polymer but also the other polymers which suffer with low FF, if a strategy can improve FF effectively. A solution‐processable small molecule BDT‐3T‐CNCOO, which contains the same donor unit as PBDTTT‐C‐T (**Figure** **1**), was combined to improve the performance of PBDTTT‐C‐T‐based OSCs. The energy levels of the components used in this work were measured by electrochemical cyclic voltammetry under similar measurement conditions (see the Supporting Information, Figure S1).^30^ Figure 1b displays the frontier level diagrams of BDT‐3T‐CNCOO, PBDTTT‐C‐T, and PC_71_BM.^31^ The cascade‐like energy levels of ternary blend OSCs may facilitate charge transfer at a donor/acceptor interface.^24^, ^26^, ^31^ Optical properties were investigated by UV–vis absorption spectrometry (Figure 1c and Figure S2 in the Supporting Information). BDT‐3T‐CNCOO showed maximum absorption at 500 nm in a chloroform solution. BDT‐3T‐CNCOO showed a redshift of 70 nm from solution to film and a new shoulder appeared in a region with a longer wavelength, which indicates the formation of a well‐ordered structure in the solid state.^32^ The maximum absorption coefficient of BDT‐3T‐CNCOO film at 570 nm is 7.6 × 10^4^ cm^−1^, whereas the absorption coefficient of PBDTTT‐C‐T at 710 nm is 4.4 × 10^4^ cm^−1^. BDT‐3T‐CNCOO is expected to compensate for the light absorption of PBDTTT‐C‐T at the short wavelength. An increase in the content of BDT‐3T‐CNCOO in the PBDTTT‐C‐T:PC_71_BM host blend largely enhanced the absorption from 400 to 640 nm (Figure 1c); by contrast, absorption gradually decreased from 640 to 760 nm.

### 2.2 Photovoltaic Properties

An inverted device structure, ITO/ZnO(≈30 nm)/BDT‐3T‐CNCOO:PBDTTT‐C‐T:PC_71_BM/MoO*_x_* (≈5 nm)/Ag(140 nm), was used in this work. The inverted structure employs good transmittance of ZnO layer (Figure S3, Supporting Information) and long‐term ambient stability of OSC devices.^10^, ^33^ A ZnO electron extraction layer was obtained by sol–gel method.^34^ After the active layer was spin‐coated, an MoO*_x_* hole extraction layer and an Ag electrode were vacuum‐deposited. The weight ratio of total donor components:PC_71_BM was maintained at 1:1.5. The weight ratio of BDT‐3T‐CNCOO in donor was changed from 0% to 100%. 1, 8‐Diiodooctance (DIO) was used as a processing additive to optimize OSC performance by promoting nanoscale phase separation.^8^ Chloroform was used as a solvent because BDT‐3T‐CNCOO was insoluble in dichlorobenzene. For the purpose of comparison, the active layers were spin‐coated at the same total concentration of donor material and at the same spin‐coating speed. The active layers exhibited a thickness of ≈105 nm.

**Figure 2:**
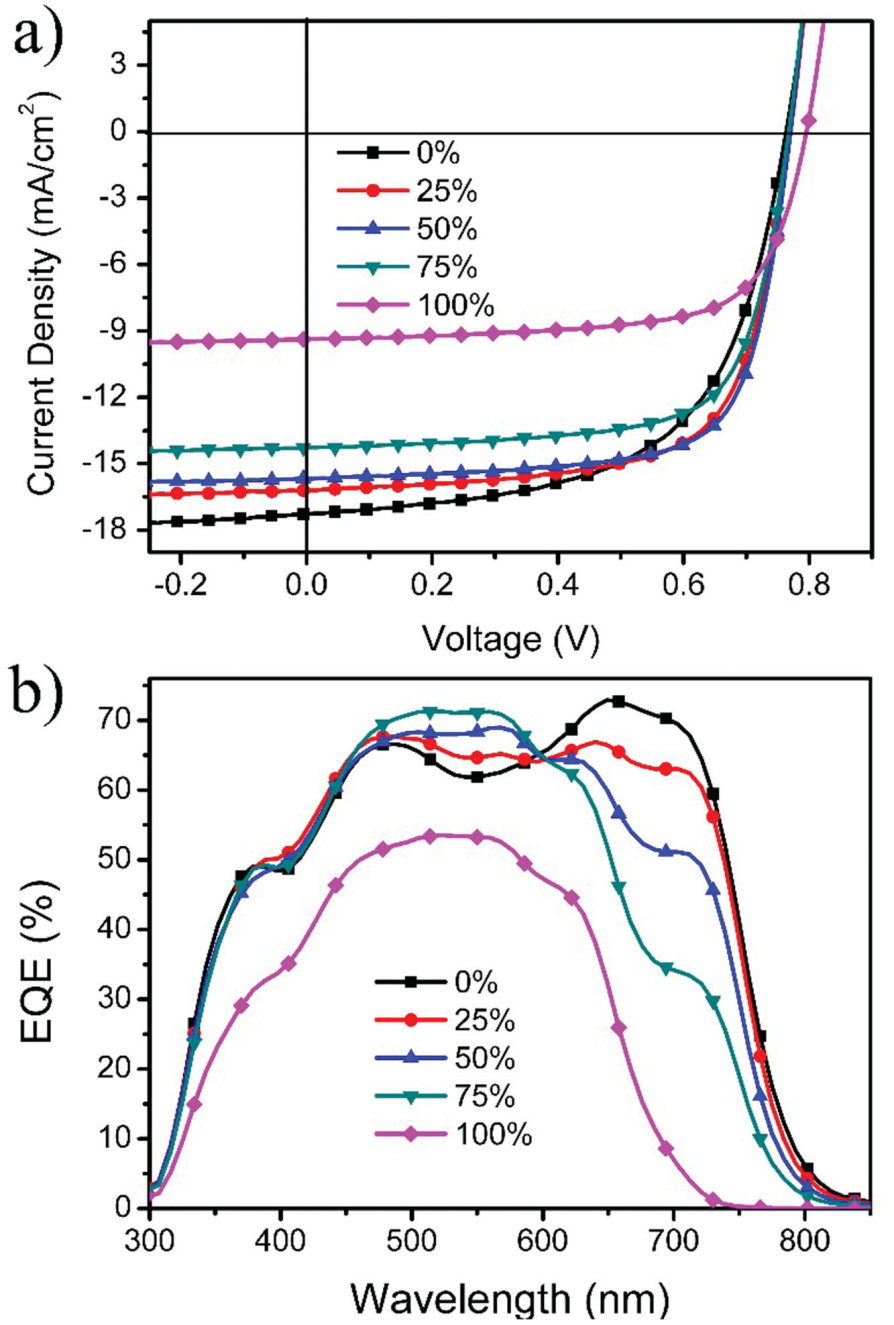
a) *J*–*V* curves of the ternary OSCs with different weight ratios of BDT‐3T‐CNCOO under A.M. 1.5G irradiation (100 mW cm^−2^); b) EQE curves of the ternary OSCs corresponding to the devices.

**Figure** **2**a shows current density versus voltage (*J–V*) characteristics of BDT‐3T‐CNCOO:PBDTTT‐C‐T:PC_71_BM devices with 0%, 25%, 50%, 75%, and 100% of BDT‐3T‐CNCOO weight contents under AM 1.5 G illumination. **Table** **1** summarizes the device performance of the ternary blend OSCs. The binary device PBDTTT‐C‐T:P_71_BM yields a PCE of 7.60% with an open circuit voltage (*V*_oc_) of 0.76 V, a short circuit current density (*J*_sc_) of 17.1 mA cm^−2^, and an FF of 58.4%. The ternary devices present the highest performance at 50% weight ratio of BDT‐3T‐CNCOO, which exhibits an average PCE as high as 8.58%, with a *J*_sc_ of 15.6 mA cm^−2^, a *V*_oc_ of 0.77 V, and an FF of 71.6%. The improvement of the PCE of the best device is mainly attributed to the remarkable improvement of FF. Compared with the best result of PBDTTT‐C‐T reported in the literature,^14^ the efficiency of our ternary blend OSCs is worse. The main reason is the lower current density of the ternary blend OSCs compared with that in the best system in which PFN interlayer was used to simultaneously improve the *V*_oc_, *J*_sc_, and *FF*. The external quantum efficiency (EQE) spectra of the corresponding devices were measured to investigate the reason for the lower current density and the trends of *J*_sc_ of the ternary blend OSCs with different ratio of BDT‐3T‐CNCOO (Figure 2b). As BDT‐3T‐CNCOO content is increased, EQEs increase between 400 and 600 nm, whereas EQEs decrease between 600 and 760 nm. A decrease in EQE is attributed to a low absorption coefficient of the polymer and a decreased ratio in the film. Overall, an increase in EQE cannot compensate for this decrease between 600 and 800 nm; as a consequence, current density decreases. However, EQEs at 710 nm of 25%, 50%, and 75% BDT‐3T‐CNCOO weight ratio devices are 92%, 75%, and 49% of EQEs of PBDTTT‐C‐T:PC_71_BM binary blend device, respectively; this result indicates that BDT‐3T‐CNCOO incorporation benefits exciton separation and charge carrier transport.

**Table 1:** Photovoltaic properties (average data of ten devices) of the ternary OSCs with different BDT‐3T‐CNCOO weight ratios under illumination of AM 1.5 G and100 mW cm^−2^

BDT‐3T‐CNCOO Ratio	Thickness [nm]	*V*_oc_ [V]	*J*_sc_ [mA cm^−2^]	FF [%]	PCE [%]
0%	104	0.76	17.1	58.4	7.60
25%	108	0.76	16.4	67.0	8.37
50%	103	0.77	15.6	71.6	8.58
75%	107	0.76	14.4	69.3	7.59
100%	104	0.79	9.2	69.0	4.98

**Figure 3:**
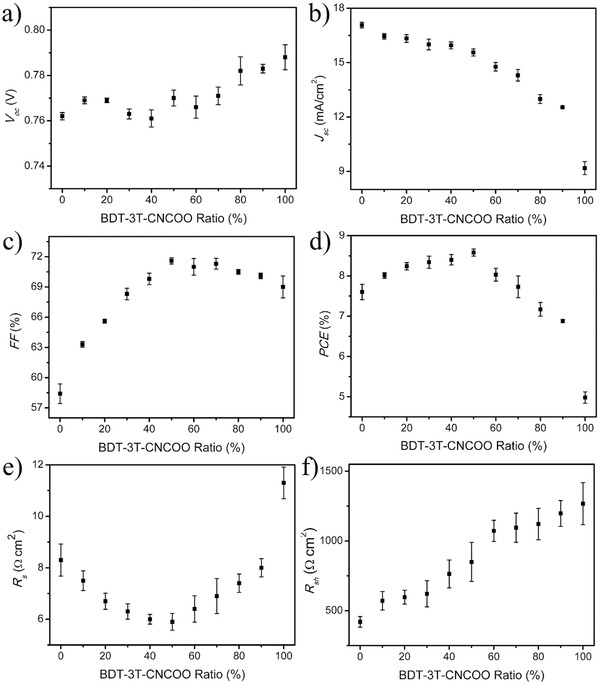
Photovoltaic properties of the ternary OSCs with different BDT‐3T‐CNCOO weight ratios. a) *V*_oc_; b) *J*_sc_; c) FF; d) PCE; e) *R*_s_; f) *R*_sh_.

The change in various key parameters, including *V*_oc_, *J*_sc_, FF, PCEs, series resistance (*R*_s_), and shunt resistance (*R*_sh_) was carefully investigated by changing the weight ratios of BDT‐3T‐CNCOO (**Figure** **3**, Table S1 in the Supporting Information) to understand the working mechanism of the ternary OSCs. *V*_oc_ of the ternary blend OSC is between binary OSCs based on small molecules (0.79 V) and polymers (0.76 V). *V*_oc_ increases as the weight ratio of BDT‐3T‐CNCOO increases. *J*_sc_ decreases slowly from 17 mA cm^−2^ to 15 mA cm^−2^ when the polymer is dominated in the ternary blend system (ratios of BDT‐3T‐CNCOO were >50%) and then decreases rapidly from 15 mA cm^−2^ to 9 mA cm^−2^ with a further increase in BDT‐3T‐CNCOO ratios. The most remarkable improvement in the photovoltaic parameters is FF. FF increases significantly from >60% in PBDTTT‐C‐T binary devices to >70% in the ternary devices when the ratio of BDT‐3T‐CNCOO is increased to 50%. PCE reaches a maximum value at 50% BDT‐3T‐CNCOO weight ratio because of the improved FF. The improved FF is attributed to low *R*_s_ and high *R*_sh_.^35^] *R*_s_ initially decreases as BDT‐3T‐CNCOO weight ratio decreases when the polymer is the dominant component; afterward, *R*_s_ increases as BDT‐3T‐CNCOO weight ratio increases when the small molecule dominates (Figure 3e). The bulk resistance of the blend layer and the contact resistance are two of the main contributions to *R*_s_.^35^, ^36^ This result indicates that the incorporated small molecule affects the charge transport property in the blend layer and the contact property between an active layer and an electrode. Figure 3f shows that *R*_sh_ almost increases linearly as the ratio of BDT‐3T‐CNCOO increases. A higher *R*_sh_ likely prevents current leakage, which indicates impurities and defects in ternary blend layer are possibly suppressed; furthermore, the interfacial morphological characteristic of a ternary blend layer is optimized.^35^, ^37^

### 2.3 Bimolecular Recombination Dynamics and Charge Mobility

**Figure 4:**
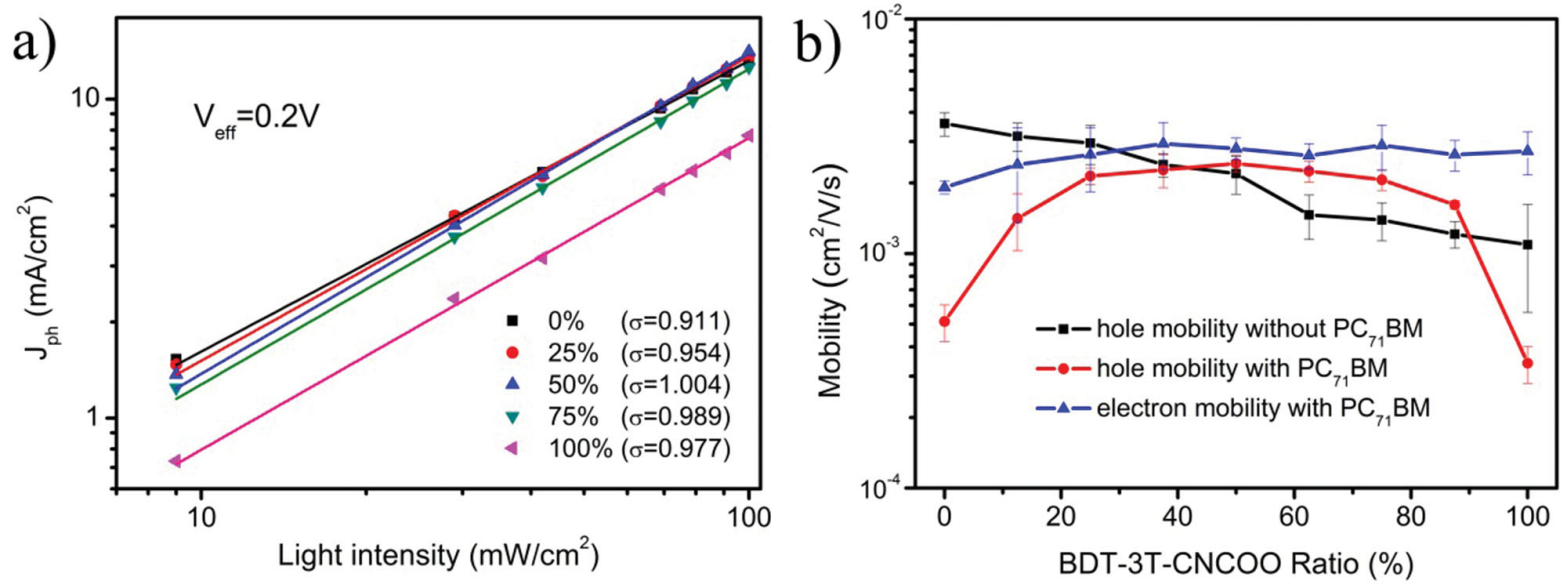
a) Photocurrent density dependence on incident light intensity of ternary blend OSCs measured under effective voltage of 0.2 V. The lines represent the fitting results. b) Hole and electron mobilities with different BDT‐3T‐CNCOO weight ratios.

We plotted photocurrents (*J*_ph_) as a function of illumination intensity (*I*) to elucidate charge recombination kinetics and to obtain further insights into exciton separation and charge transfer of ternary blend OSCs; we then determined hole mobilities of PBDTTT‐C‐T:BDT‐3T‐CNCOO, hole mobilities and electron mobilities of PBDTTT‐C‐T:BDT‐3T‐CNCOO:PC_71_BM film with different ratios of BDT‐3T‐CNCOO by space charge limited current method. **Figure** **4**a shows the dependence of *J*_ph_ at 0.2 V effective voltage on light intensity on a log–log scale and the data are fitted by a power law *J*_sc_ ∝ *I^σ^*. The fitted slopes (*σ*) of ternary blend devices with 0%, 25%, 50%, 75%, and 100% of BDT‐3T‐CNCOO are about 0.911, 0.954, 1.004, 0.989, and 0.977, respectively. This result shows that all of the devices exhibit weak bimolecular recombination.^37^, ^38^ However, as the blend of BDT‐3T‐CNCOO, the bimolecular recombination of ternary blend devices is weaker than that of binary devices. Especially, for 50% BDT‐3T‐CNCOO ternary blend device which has the best performance due to the weakest bimolecular recombination.

Figure 4b shows the hole and the electron mobilities calculated by fitting the *J–V* characteristics of single carrier diodes (Figure S4 Supporting Information) with Mott–Gurney law. We found that hole mobilities of PBDTT‐C‐T:BDT‐3T‐CNCOO films decrease almost linearly with the ratio of BDT‐3T‐CNCOO. Blending PC_71_BM shows a negative effect on hole mobilities of binary blend films, indicating the blend of PCBM disrupting the structure of the donor films. Interesting, compared with pristine donor films, hole mobilities of the ternary blend films are higher than those of binary blend films, suggesting that phase behavior of donors in ternary blend films is different from that of the binary films. However, the electron mobilities almost keep constant between ≈2 × 10^−3^ and 3 × 10^−3^ cm^2^ V^−1^ s^−1^. The highest hole mobility and the most balanced charge transport are achieved at 50% ratio of BDT‐3T‐CNCOO. The increased and more balanced charge mobilities facilitate the charge sweep‐out in the active layer.^35^ As a consequence, the FF of ternary devices is improved because of weak carrier recombination and easier charge sweep‐out.^23^

### 2.4 Film Morphology, Crystallinity, and Phase Separation

Tapping model atomic force microscopy (AFM) was applied to investigate the surface morphological characteristic of ternary blend films. The height images are shown in Figure S5 in the Supporting Information. The root‐mean‐square (RMS) roughness values of 0%, 25%, 50%, 75%, and 100% weight ratios of BDT‐3T‐CNCOO blend films are 2.10, 2.68, 4.51, 4.56, and 5.42 nm, respectively. The incorporation of BDT‐3T‐CNCOO in the blend film increases surface roughness. The increased roughness of the films possibly introduces greater contact resistance and causes an increase in *R*_s_ when the weight ratio of BDT‐3T‐CNCOO exceeds 50%. Based on the AFM images, the aggregation on the surface of ternary blend films is similar to that of BDT‐3T‐CNCOO:PC_71_BM binary blend films; this finding indicates that the small molecule likely aggregates on the surface. Donor‐enriched layers can function as an electron‐blocking region, which suppresses electron leakage to an MoO*_x_*‐coated anode; thus, recombination at contacts is reduced.^39^ It might be a new strategy to modulate surface composition, as it should be considered to get higher efficient OSCs.^14^ The inverted structure performance is compared with that of conventional structure (Table S2, Supporting Information), and the result further supports vertical phase segregation.

**Figure 5:**
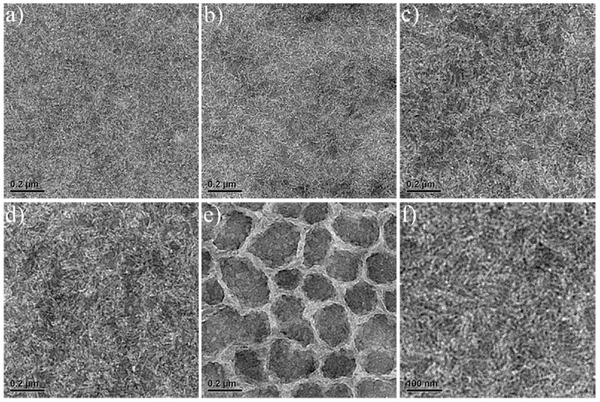
TEM images of the active layer with different BDT‐3T‐CNCOO weight ratios: a) 0%; b) 25%; c) 50%; d) 75%; e) 100%; f) 50%, respectively (scale bar = 100 nm).

**Figure** **5** shows the TEM images of ternary blend films. The bright and dark regions in TEM images are normally assigned as donor‐rich and PCBM‐rich domains, respectively.^42^ The phase separation of PBDTTT‐C‐T:PC_71_BM binary blend is not obvious, but nanofibrous structures of PBDTTT‐C‐T with diameters of tens of nanometers can be observed. The unobvious phase separation does not facilitate the charge sweep‐out, which is consistent with the low FF of PBDTTT‐C‐T:PC_71_BM binary device. On the other hand, the domain size of BDT‐3T‐CNCOO:PC_71_BM binary blend film reaches ≈100 nm. Such a big domain size is unmatched with an exciton diffusion length of ≈10 nm, which is consistent with the low *J*_sc_ of BDT‐3T‐CNCOO:PC_71_BM binary device. The ternary blend films show obvious nanofibrous feature with diameters of tens of nanometers, indicating enhanced phase separation.^40^, ^41^ The increased domain size and the nanofibrous feature are advantageous to form an effective continuous interpenetrating D–A charge transport pathway. The large domain size might be attributed to the high crystallinity of the small molecule. A nanofibrous feature is clearly observed in a large donor domain (Figure S6, Supporting Information) when ≈10% PBDTTT‐C‐T is blended into BDT‐3T‐CN‐COO:PC_71_BM system. This finding indicates that BDT‐3T‐CNCOO and PBDTTT‐C‐T exhibit a synergistic effect, that is, BDT‐3T‐CNCOO serves as a driving force of PBDTTT‐C‐T to form a fibrous structure and PBDTTT‐C‐T restrains BDT‐3T‐CNCOO to form a large aggregate structure; thus, an optimal domain size can be formed in a ternary blend film.

**Figure 6:**
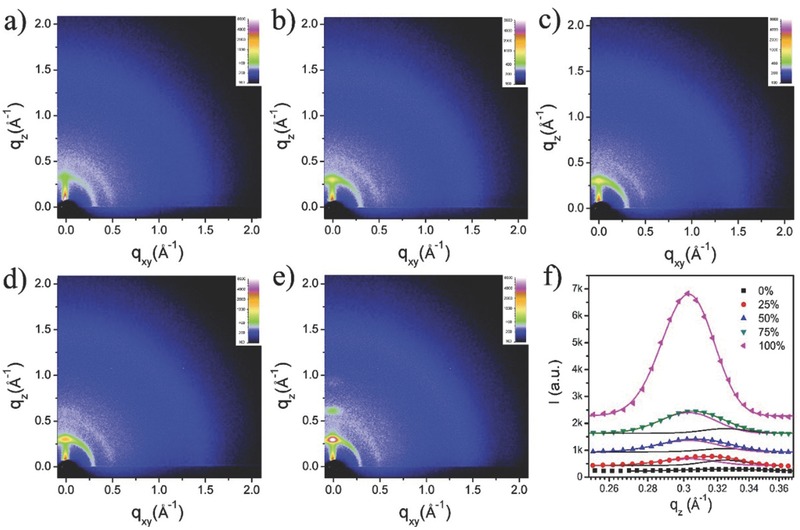
2D GIWAXS images of the active layer with different BDT‐3T‐CNCOO weight ratios: a) 0%; b) 25%; c) 50%; d) 75%; e) 100%, respectively. f) Fitting the out‐of‐plane lamellar stacking peaks by using Gaussian function.

GIWAXS measurement was performed to investigate the crystalline structure of ternary blend films.^43^ The 2D GIWAXS patterns obtained from spin‐casted films with different weight ratios of BDT‐3T‐CNCOO are shown in **Figure** **6**a–e. The PC_71_BM aggregation peak at about 1.35 Å^−1^ in both directions is similar with each other, which indicates that the introduction of BDT‐3T‐CNCOO in the ternary blend film does not influence the aggregation of fullerenes. And it is consistent with the small variation of electron mobilities. The π–π stacking (010) peak at ≈1.8 Å^−1^ could be observed in both directions (Figure S7a, Supporting Information), however, the intensity of the peak is very weak. The presence of lamellar (h00) stacking peaks is attributed to the alkyl chain distance between neighboring backbones (*d*‐spacing) in an out‐of‐plane direction (Figure S7a, Supporting Information). The lamellar stacking (100) peaks of PBDTTT‐C‐T and BDT‐3T‐CNCOO are located at 0.325 and 0.303 Å^−1^, respectively, corresponding to *d*‐spacing of 19.3 and 21.8 Å. PBDTTT‐C‐T and BDT‐3T‐CNCOO lamellar stacking peaks can be distinguished in the ternary blend films based on the difference in the *d*‐spacing distance by using Gaussian function to fit the peaks; thus, the (100) peak of the out‐of‐plane profile in Figure S7a in the Supporting Information is fitted by two peaks, which are located at the corresponding pure PBDTTT‐C‐T and BDT‐3T‐CNCOO, respectively (Figure 6f). Based on the full width at half maximum of the fitted peaks, the out‐of‐plane lamellar stacking coherence lengths of PBDTTT‐C‐T and BDT‐3T‐CNCOO can be calculated by Scherrer equation.^44^ The coherence lengths along the lamellar stacking direction of ternary blend films with 0%, 25%, 50%, 75%, and 100% of BDT‐3T‐CNCOO are plotted in Figure S7b in the Supporting Information. In the polymer, the coherence lengths of 0%, 25%, 50%, and 75% BDT‐3T‐CNCOO ratio ternary blend films are 10.6, 18.3, 18.1, and 19.6 nm, respectively; this result suggests that the crystalline structure of PBDTTT‐C‐T is enhanced when a small molecule is added. The coherence lengths of the small molecule are 14.4, 14.4, 15.7, and 16.6 nm in 25%, 50%, 75%, and 100% BDT‐3T‐CNCOO ratio blend films, respectively. This finding indicates that PBDTTT‐C‐T restrains the crystalline structure of BDT‐3T‐CNCOO, which is consistent with the TEM image shown in Figure S6 in the Supporting Information.

**Figure 7:**
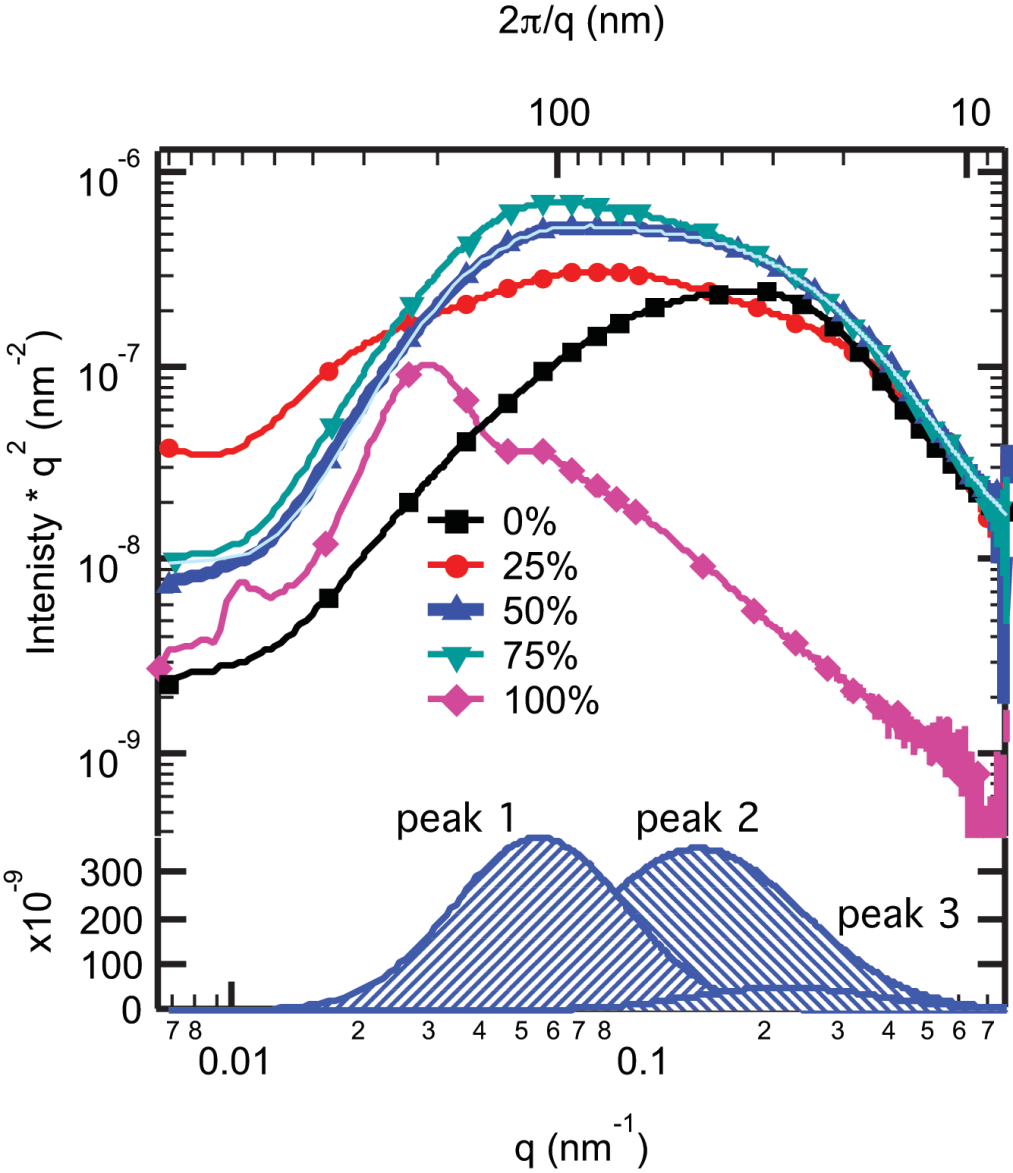
R‐SoXS scattering profiles at 284.2 eV of ternary and binary blends with different small molecular ratios.

The phase separation of the ternary blends is further investigated by R‐SoXS. Based on enhanced contrast among different components, the characteristic median length (domain spacing) and average compositional fluctuation (domain purity) can be obtained.^46^, ^47^, ^48^, ^49^, ^50^, ^51^ **Figure** **7** shows the scattering profile of ternary blends and binary reference blend at 284.2 eV. Scattering can be fitted with a set of log‐normal distributions (Figure S8, Supporting Information).^48^ The scattering of 100% BDT‐3T‐CNCOO is not fitted because of strong structure factor that can be inferred from TEM image. With this novel analysis, we can obtain phase separation at different length scales. The fitting of 50% BDT‐3T‐CNCOO and its components is plotted in Figure 7 as an example. Multiple peaks indicate that phase separations are present at multiple length scales in the ternary blends; this finding is consistent with the TEM results shown in Figure 5e,f. The location of q_2_ and q_3_ in the ternary blends is almost similar to the peak of PBDTTT‐C‐T:PC_71_BM reference binary and remains unchanged with composition; this result indicates that these peaks correspond to the polymer/fullerene phase separation in the ternary blend. The origin of q_1_ should only be BDT‐3T‐CNCOO:PC_71_BM phase separation (but not small molecule and polymer phase separation) because of the low scattering contrast of BDT‐3T‐CNCOO:PBDTTT‐C‐T. The log‐normal distributions exhibit a characteristic median length scale *ξ*_med_ = 2*π*/*q*_med_. As BDT‐3T‐CNCOO ratio changes, the phase separation of PBDTTT‐C‐T:PC_71_BM yields *ξ* of ≈22 nm. The phase separations of BDT‐3T‐CNCOO:PC_71_BM *ξ* are 65, 88, 90, and 200 nm when 25%, 50%, 75%, and 100% BDT‐3T‐CNCOO are added. This result suggests that an increase in BDT‐3T‐CNCOO loading likely induces large BDT‐3T‐CNCOO:PC_71_BM phase separation. The larger domain is unfavorable for charge separation efficiency. This finding is another factor accounted for the decrease in *J*_sc_ */*EQE as BDT‐3T‐CNCOO content in ternary blends decreases.

Furthermore, the total scattering intensity (TSI, obtained by integrating the scattering profiles) can be calculated to describe the relative domain purity of the blends.^48^, ^52^ The relative purities of PBDTTT‐C‐T:PC_71_BM domain (sum TSI of peak 2 and peak 3) are 83%, 85%, 99%, and 100% when 0%, 25%, 50%, and 75% BDT‐3T‐CNCOO are used, suggesting that PBDTTT‐C‐T:PC_71_BM domain becomes purer when additional small molecules are added. The relative purities of BDT‐3T‐CNCOO:PC_71_BM domain are 46%, 65%, 100%, and 30% when 25%, 50%, 75%, and 100% BDT‐3T‐CNCOO are used. This result indicates that BDT‐3T‐CNCOO:PC_71_BM domains are also purified when more BDT‐3T‐CNCOO is loaded. However, 100% BDT‐3T‐CNCOO yields a relative mixed domain. All of the ternary blends exhibit purer BDT‐3T‐CNCOO:PC_71_BM and PBDTTT‐C‐T:PC_71_BM domains than the corresponding binary blend. Purer domain results in a reduced bimolecular recombination.^47^, ^53^ This finding also explains why ternary blends show higher hole mobility and FF than binary blends. Overall, R‐SoXS data show that hierarchical phase separation composed of tunable BDT‐3T‐CNCOO:PC_71_BM domain size and more pure PBDTTT‐C‐T:PC_71_BM domain forms in the ternary blend films. The hierarchical phase separation is a favorable morphology for the BHJ OSCs, which provide a maximum interface area for efficient dissociation and continuous pathway for charge transport and collection.^14^, ^54^, ^55^

## 3 Conclusions

In summary, inverted ternary OSCs based on soluble small molecule and low band gap polymer were successfully fabricated. The PCE of the device prepared at the optimized ratio of BDT‐3T‐CNCOO:PBDTTT‐C‐T:PC_71_BM reached 8.58%. The evolution of the morphological characteristics, crystalline structure, and phase separation from a PBDTTT‐C‐T:PC_71_BM binary blend film to BDT‐3T‐CNCOO:PBDTTT‐C‐T:PC_71_BM ternary blend films was determined by AFM, TEM, GIWAXS, and R‐SoXS measurement. Our results showed that the high crystallinity of BDT‐3T‐CNCOO likely provides the driving force of PBDTTT‐C‐T to self‐assemble to a fibrous structure with larger crystal size and greater purity domains in a ternary blend film; by contrast, a long‐chain PBDTTT‐C‐T possibly restrains the crystalline structure of BDT‐3T‐CNCOO and an unfavorable aggregation of small molecule/fullerene domains. Hierarchical phase separation forms in the ternary blend films, which balances the charge separation and transport. Therefore, the FF of the ternary OSCs can be improved effectively. On the basis of ideal morphological characteristics, improved charge mobilities, and suppressed charge recombination, we found that the FFs of OSCs were enhanced by ≈20% compared with those of a PBDTTT‐C‐T‐based binary system.

## 4 Experimental Section

*Fabrication of the Ternary OSCs*: PBDTTT‐C‐T (Solarmer Energy, Inc.) and PC_71_BM (American Dye Source, Inc., 99.5%) were used as received. BDT‐3T‐CNCOO was synthesized in our lab. Prepatterned indium–tin oxide (ITO)‐coated glass with a sheet resistance of 15 Ω sq^−1^ was used as a substrate after being cleaned by successive sonication in deionized water, ethanol, acetone, and isopropanol thrice for 10 min. ITO‐coated glass was dried by compressed N_2_ gas. After 5 min of ultraviolet/ozone treatment (Jelight), a ZnO layer of approximately 30 nm was prepared by spin‐coating (at 3000 rpm) a precursor solution prepared from 0.45 m zinc acetate dehydrate in 0.45 m ethanolamine and 2‐methoxy ethanol. After the electrical contact was cleaned, ZnO‐coated ITO glass substrates were immediately baked in air at 200 °C for 30 min. The substrates were then transferred into a nitrogen‐filled glove box. PBDTTT‐C‐T:BDT‐3T‐CNCCOO:PC_71_BM was dissolved in chloroform and spin‐coated at 3000 rpm on the top of the ZnO layer. The overall donor concentration was 10 mg mL^−1^ and the D/A ratio was maintained at 1:1.5. MoO*_x_* (5 nm) and Ag (140 nm) were then thermally evaporated by a shadow mask at approximately 2 × 10^−6^ mbar. The active area of the OSC devices was 0.04 cm^2^.

*Measurements and Characterization*: Photovoltaic performance was determined using a Keithley 2400 source meter at AM 1.5 G (100 mW cm^−2^) simulated by a Newport solar simulator. The light intensity was calibrated using an Si diole and a light source meter before measurement was conducted. EQEs were characterized using an Oriel QE/IPCE measurement kit (Model QE‐PV‐SI). TEM measurements were performed using a Tecnai G2 U‐TWIN (FEI) transmission electron microscope under proper defocus conditions. To prepare the specimens, we floated the blend films onto the surface of deionized water and then transferred the films to copper grids. GIWAXS measurements were performed in a Xeuss SAXS/WAXS system with a wavelength of *λ*= 1.5418 Å. R‐SoXS measurements were performed at a beamline 11.0.1.2 (Advanced Light Source, Lawrence Berkeley National Laboratory, Berkeley, CA).^56^

## Supporting information

As a service to our authors and readers, this journal provides supporting information supplied by the authors. Such materials are peer reviewed and may be re‐organized for online delivery, but are not copy‐edited or typeset. Technical support issues arising from supporting information (other than missing files) should be addressed to the authors.

Supplementary
