# Supplementary material for: Understanding the Impact of Hierarchical Nanostructure in Ternary Organic Solar Cells
Source: Adv Sci (Weinh). 2015 Sep 2;2(10):1500250. doi: 10.1002/advs.201500250 (PMC5049664; doi:10.1002/advs.201500250)
Supplement: Supplementary file 1 — Supplementary [file ADVS-2-0f-s001.pdf]

Copyright WILEY-VCH Verlag GmbH & Co. KGaA, 69469 Weinheim, Germany, 2015.

ADVANCED SCIENCE
Open Access

Supporting Information

for *Adv. Sci.*, DOI: 10.1002/advs.201500250

Understanding the Impact of Hierarchical Nanostructure in Ternary Organic Solar Cells

Jin Fang, Zaiyu Wang, Jianqi Zhang, Yajie Zhang, Dan Deng, Zhen Wang, Kun Lu, Wei Ma,* and Zhixiang Wei**

WILEY-VCH

Copyright WILEY-VCH Verlag GmbH & Co. KGaA, 69469 Weinheim, Germany, 2013.

Supporting Information

Understanding the Impact of Hierarchical Nanostructure in Ternary Organic Solar Cells

Jin Fang, Zaiyu Wang, Jianqi Zhang, Yajie Zhang, Dan Deng, Zhen Wang, Kun Lv, Wei Ma*, Zhixiang Wei**

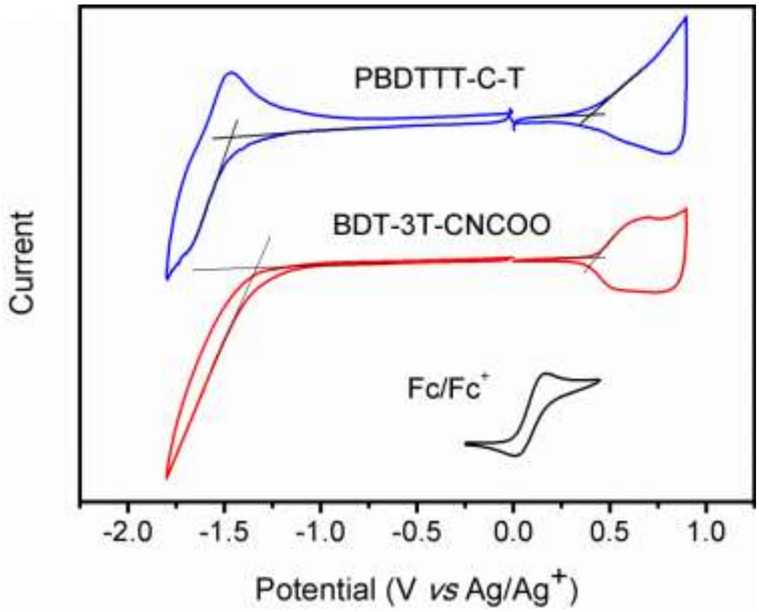

Figure S1. Cyclic Voltammograms of BDT-3T-CNCOO and PBDTTT-C-T thin films.

The electrochemical properties of the donors were investigated as thin film in anhydrous acetonitrile under N_2 at a scan rate of 50 mV/s using 0.1M tetrabutylammonium hexafluorophosphate $[(n\text{-Bu})_4N^+PF_6^-]$ as supporting electrolyte, Pt electrodes were used as both the working and the counter electrodes, and Ag/Ag^+ was used as the reference electrode. For calibration, the ferrocene/ferrocenium redox couple was measured under the same condition, and the formal potential is located at 0.09V to the Ag/Ag^+ electrode. It is assumed that the redox potential of Fc/Fc^+ has an absolute energy level of -4.80 eV to vacuum. The energy levels of the highest occupied (HOMO) and lowest unoccupied molecular orbital (LUMO) were then calculated according to the following equation:

$$\mathrm{HOMO} = -\mathrm{e}(E_{ox} + 4.71V)$$

$$\mathrm{LUMO} = -\mathrm{e}(E_{red} + 4.71V)$$

where E_{ox} is the onset of oxidation potential versus Ag/Ag^+ and E_{red} is the onset of reduction potential versus Ag/Ag^+.

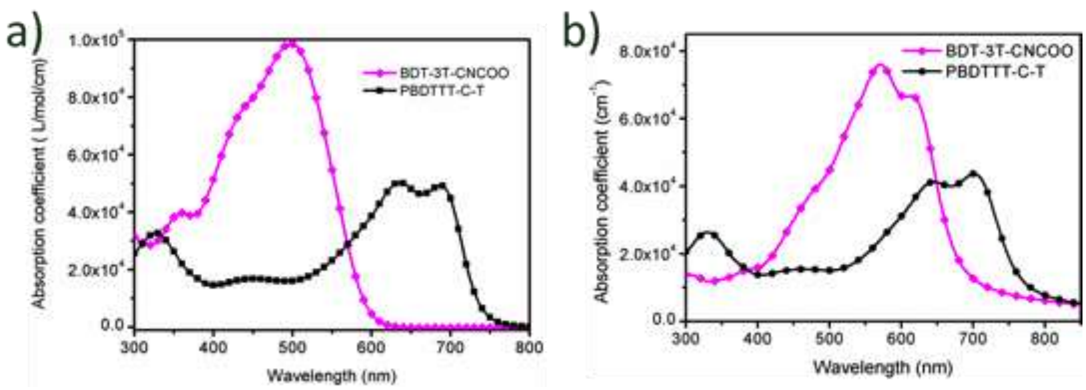

Figure S2. UV-vis absorption spectra of BDT-3T-CNCOO and PBDTTT-C-T a) in solution; b) on film.

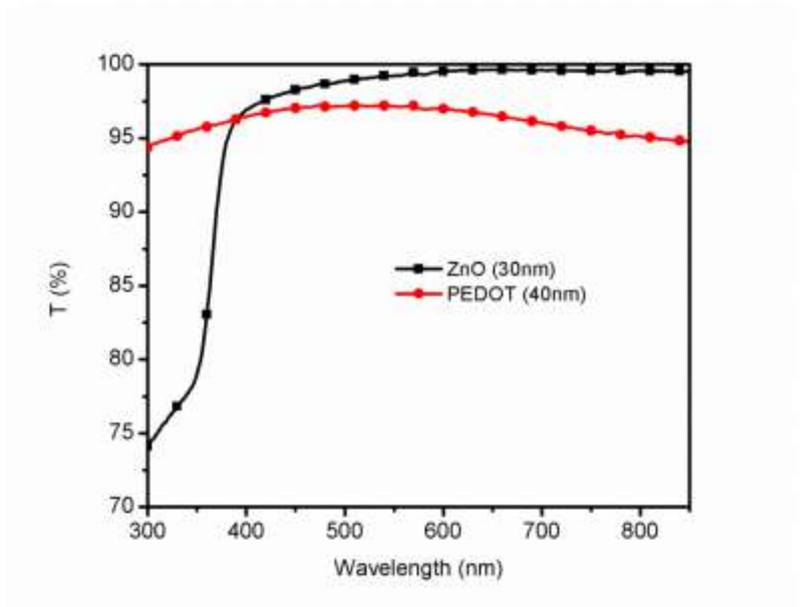

Figure S3. Transmittance spectrum of ZnO (*sol-gel* method, ~30nm) layer and PEDOT:PSS (Baytron P VPAI 4083, ~40nm) on quartz sheet.

UV-vis absorption spectra were record on a Shimadzu UV3600 UV-vis-NIR spectrophotometer. The solution absorption spectra of the donors were recorded on 10^{-5}M (based on repeating units for the polymer) chloroform solution, and the film optical absorption spectra were recorded from films cast from 10mg/ml chloroform solutions.

Table S1. Photovoltaic properties of the ternary OSCs with different BDT-3T-CNCOO: PBDTTT-C-T weight ratio under illumination of A.M. 1.5 G, 100mW/cm^2. Performance metrics reported are the average of at least 4 devices. The active layer was spin-coated at the same total concentration and the same spin-coating speed. And the thickness of all active layer is around 105nm.

BDT-3T-CNCOO Ratio	*V_{oc} (V)*	*J_{sc} (mA/cm^2)*	*FF (%)*	*PCE (%)*	*R_s (Ωcm^2)*	*R_{sh} (Ωcm^2)*
0%	0.762±0.002	17.07±0.16	58.40±0.97	7.60±0.19	8.3±0.6	419.9±38.2
10%	0.769±0.002	16.45±0.15	63.30±0.28	8.01±0.07	7.5±0.4	571.5±66.1
20%	0.769±0.001	16.33±0.21	65.60±0.19	8.24±0.09	6.7±0.3	596.8±49.0
30%	0.763±0.002	16.00±0.30	68.30±0.57	8.34±0.15	6.3±0.3	621.0±93.6
40%	0.761±0.004	15.95±0.19	69.80±0.57	8.40±0.13	6.0±0.2	763.5±100.4
50%	0.770±0.004	15.56±0.19	71.60±0.31	8.58±0.09	5.9±0.3	849.5±139.7
60%	0.766±0.005	14.77±0.23	71.00±0.83	8.03±0.16	6.4±0.5	1072.5±75.4
70%	0.771±0.004	14.30±0.32	71.29±0.86	7.73±0.27	6.9±0.7	1095.0±104.3
80%	0.782±0.006	12.99±0.25	70.50±0.24	7.17±0.17	7.4±0.4	1120.9±112.1
90%	0.783±0.002	12.54±0.03	70.10±0.27	6.88±0.04	8.0±0.4	1197.1±92.5
100%	0.788±0.006	9.18±0.36	69.00±1.09	4.98±0.14	11.3±0.6	1267.4±150.8

Table S2. Photovoltaic properties of the ternary OSCs based on conventional device structure(ITO/PEDOT:PSS/Active Layer/Ca(10nm)/Al(100nm)) under illumination of A.M. 1.5 G, 100mW/cm^2.

BDT-3T-CNCOO Ratio	*V_{oc} (V)*	*J_{sc} (mA/cm^2)*	*FF (%)*	*PCE(PCE_{ave}) (%)*
0%	0.78	13.82	61.1%	6.59%(6.26%)
10%	0.78	14.38	64.3%	7.25%(6.97%)
20%	0.78	14.12	67.6%	7.46%(7.26%)
30%	0.79	13.90	68.6%	7.50%(7.27%)
40%	0.78	13.29	70.0%	7.22%(7.00%)
50%	0.79	13.16	69.1%	7.16%(6.97%)
60%	0.79	12.86	67.1%	6.78%(6.55%)
70%	0.79	11.91	68.5%	6.43%(6.32%)
80%	0.80	11.35	66.2%	5.99%(5.91%)
90%	0.81	10.56	65.4%	5.62%(5.32%)
100%	0.94	7.24	69.3%	4.71%(4.40%)

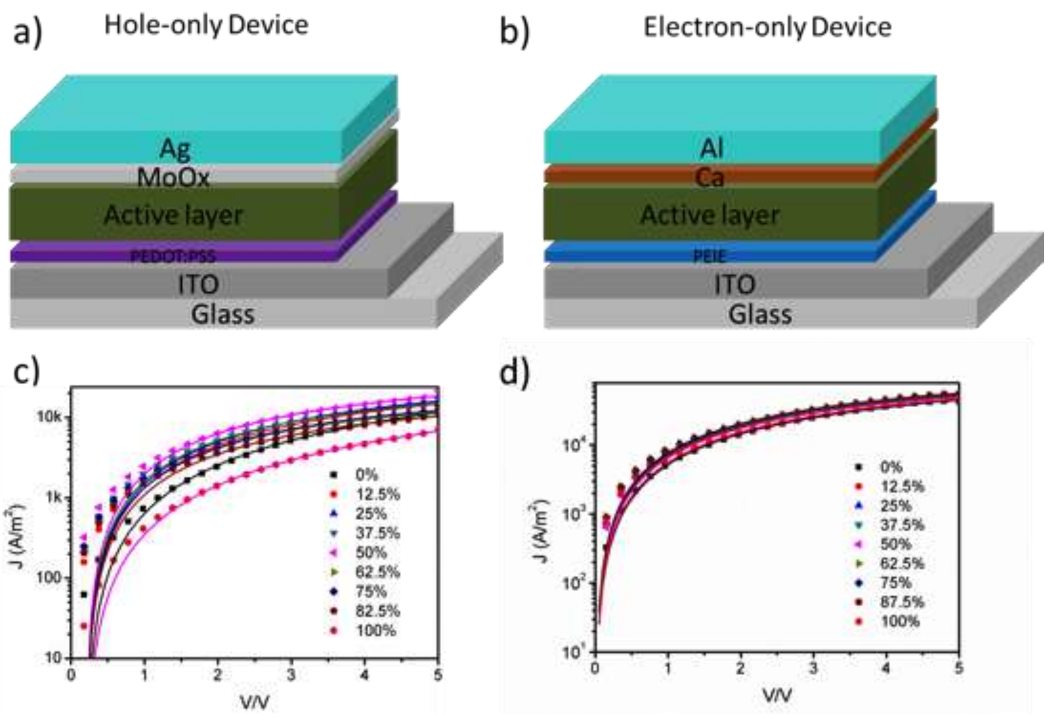

Figure S4. a) Hole-only diodes structure; b) Electron-only diodes structure; c) Typical current density-applied voltage semi-log plots for hole-only diodes; d) Typical current density-applied voltage semi-log plots for electron-only diodes. Measured data are show as symbols while the solid lines are best fits to the space-charge-limited-conduction (SCLC) model. Mobilities were extracted from the fitting.

The hole-only diode was fabricated using a device structure of ITO/PEDOT:PSS(40nm)/Active layer(~100nm)/MoO_x(5nm)/Ag(100nm). The electron-only diode was fabricated using a device structure of ITO/PEIE(5nm)/Active layer(100nm)/Ca(20nm)/Al(100nm). Polyethyleneimine ethoxylated (PEIE) was used to tune the work function of ITO. At high voltages regime, the J-V characteristics of the diodes was fitted using Mott-Gurney law, that includes field-dependent mobility, to extract the zero-field mobility μ_0. The Mott-Gurney law that includes the field-dependent mobility:

$$J = \frac{9}{8}\varepsilon_0 \varepsilon_r \mu_0 \frac{(V - V_{bi})^2}{L^3} exp\left(\beta\sqrt{\frac{V - V_{bi}}{L}}\right)$$

where ε_0 is the permittivity of free space, ε_r is the dielectric constant of the semiconductor layer, μ_0 is the zero-field mobility, V_{bi} is the build-in voltage due to the anode-cathode work function offset, L is the thickness of the active layer, and β is the field-dependence coefficient. The dielectric ε_r is assumed to be 3 and 3.9 for the hole-only and electron-only diodes, respectively. The thickness of the active layer was measured by using a Dektek profilometer.

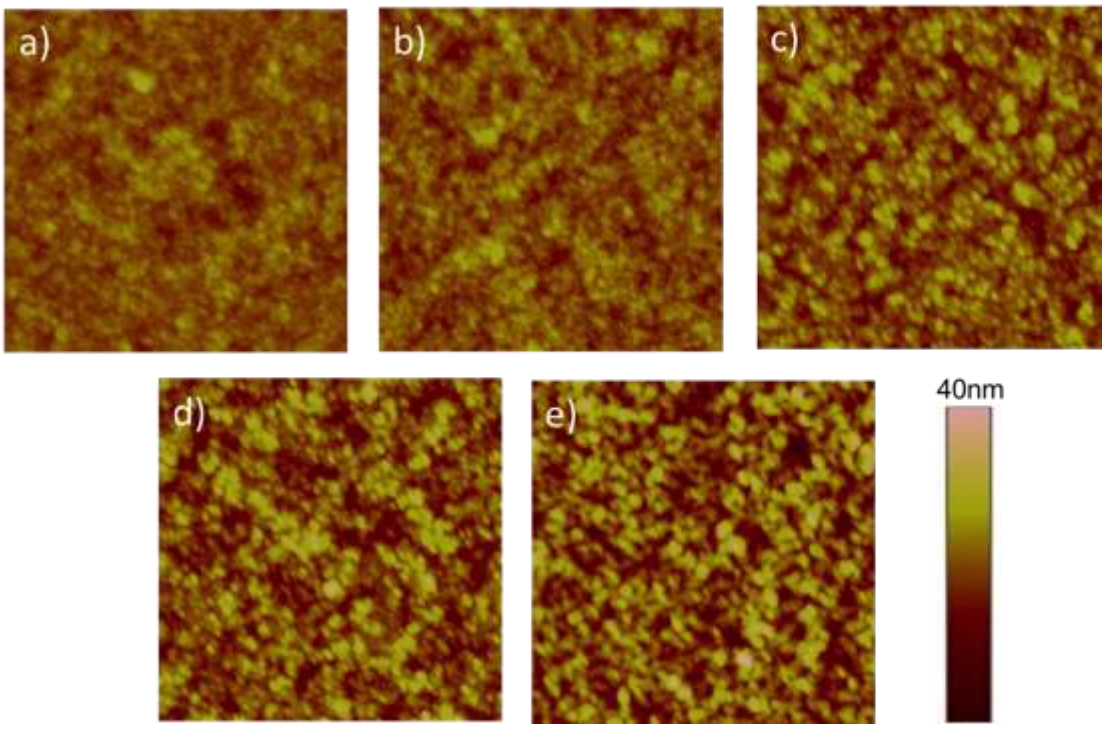

Figure S5. AFM topographical image (5μm×5μm) of the ternary OSCs blend films under different BDT-3T-CNCOO weight ratio: a) 0%, b) 25%, c) 50%, d) 75%, and e) 100%.

AFM measurements were performed using a Dimension 3100 scanning probe microscope (Veeco) in tapping mode.

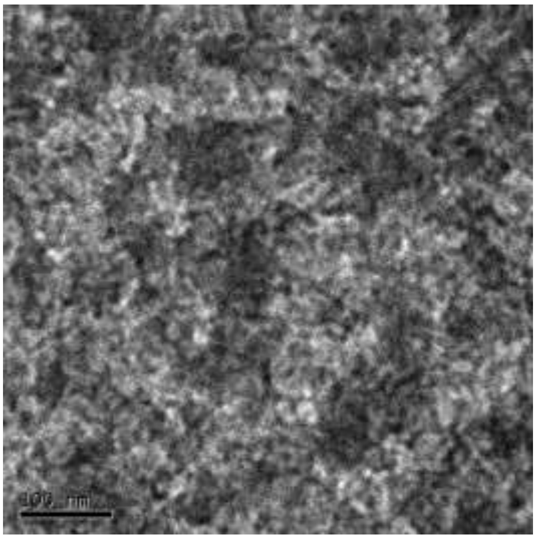

Figure S6. TEM images of the active layer with 90% BDT-3T-CNCOO weight ratio.

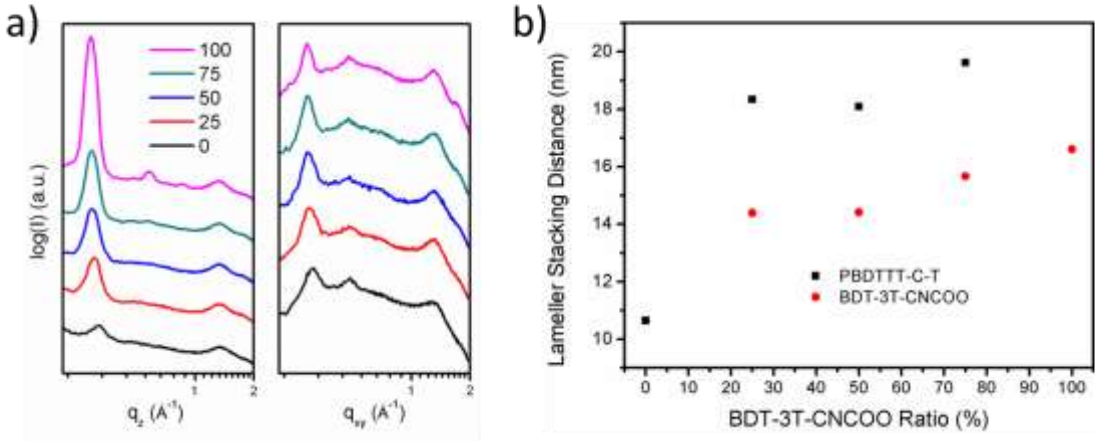

Figure S7. a) Out-of-plane and in-plane line cuts of 2D GIWAXS of the ternary blend films; b) the lamellar stacking coherence length estimated by using the full width at half-maximum (fwhm) of the fitting peaks based on the Scherrer equation.

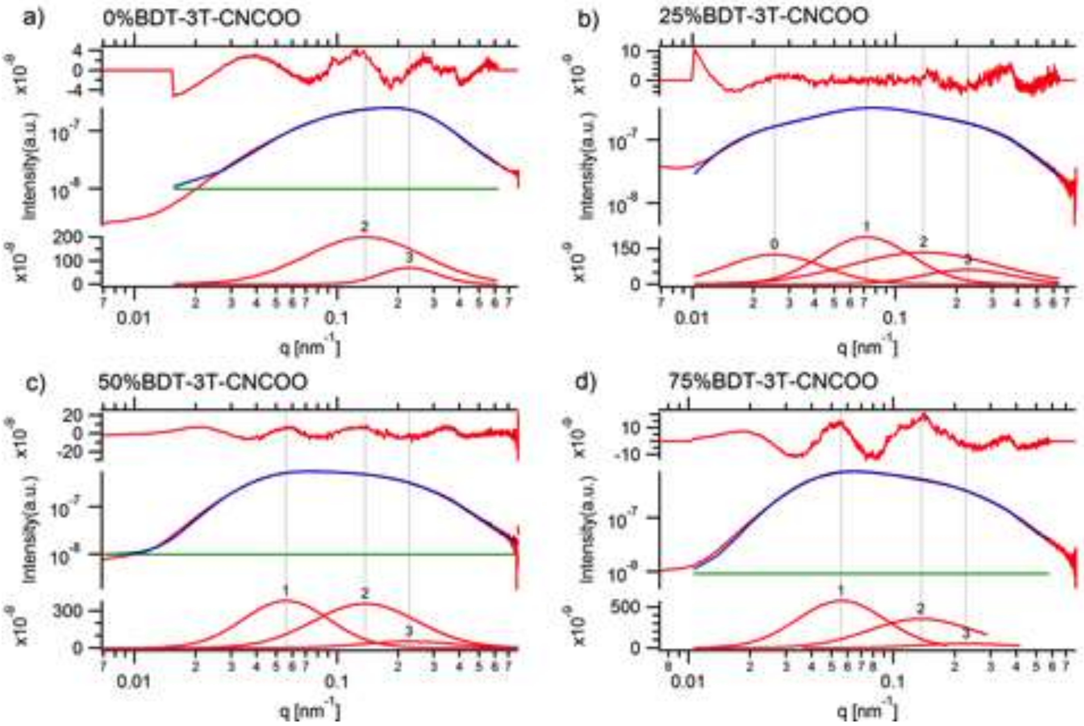

Figure S8. The R-SoXS fitting results with multi- lognormal distributions.
